# Identifying trends in nursing start-ups using text mining of YouTube content

**DOI:** 10.1371/journal.pone.0226329

**Published:** 2020-02-13

**Authors:** Ji Young Lim, Seulki Kim, Juhang Kim, Seunghwan Lee

**Affiliations:** 1 Department of Nursing, Inha University, Incheon, Korea; 2 Department of Nursing, Far East University, Chungbuk, Korea; 3 Department of Statistics, Inha University, Incheon, Korea; University of Albany, State University of New York, UNITED STATES

## Abstract

This study uses YouTube content to explore trends in nursing start-ups. YouTube content can be used to understand the current trends regarding interest and awareness in various fields. The study was conducted in three stages: text mining, Delphi survey, and comparison. The frequency and degree centrality of keywords were analyzed in the text mining stage. In the Delphi survey, the 100 most frequent keywords were classified using a synthesis framework for nursing start-ups. In the comparison stage, the results of text mining and the Delphi survey were matched using a 2x2 matrix. Text mining identified “area,” “business,” “competence,” “idea,” and “success” as the most commonly used keywords. The keywords that showed the highest level of classification agreement in Delphi were “motivation,” “advice,” “obstacle,” “business,” “charisma,” and “result.” In the comparison using a 2x2 matrix, “dream,” “idea,” “opportunity,” “leadership,” “success,” “benefit,” and “satisfaction” emerged. The results indicate that interest in nursing start-ups develops at an early stage. In order to encourage nursing start-ups, it is necessary to strengthen business skills such as finance and budgeting, establish active policy support for such start-ups, and develop new nursing start-up items appropriate for the Fourth Industrial Revolution.

## Introduction

The development of data communication technology has facilitated media fusion and has resulted in an exponential increase in the usage of various forms of social media, such as texts, images, audio, and video [1]. The widespread adoption of the internet has further increased sharing of video and image content, leading to the establishment of YouTube, a free video-sharing website, in 2005 [2]. As of May 2018, YouTube is one of the most popular user-created content platforms with 1.8 billion users [3]. YouTube’s contents can only be estimated; prior studies have shown that 19,025 channels and 5,591,400 video analyses were conducted since 2006 [4]. This vast volume of content can be shared freely without the restriction of time or space. YouTube enables users to engage and share personal opinions and information with a large number of other users [5].

YouTube content can help determine contemporary trends of interest and awareness in various fields. Gupta, Lam, Pettigrew, and Tait [6], for example, conducted research regarding two alcohol brands with each having a significant number of YouTube subscribers. The two brands originated in Australia and India, two countries with significant cultural differences. The researchers found that much of the content in Australia emphasized the tradition of the brand, while the advertisements in India were mostly of a sexual nature. A comparative analysis of YouTube content across countries indicates that having a marketing strategy tailored to the country’s cultural context is crucial. Kim, Park, and Park [7] analyzed YouTube content relating to the 2017 presidential nomination acceptance speech in Korea. They found that the proportion of positive comments for the current president, Jae-in Moon, was high, which demonstrated the potential of using YouTube analysis for future election campaigns.

As such, Big Data analysis utilizing text mining can detect shifts and trends in public thought and opinion [8]. Identifying the appearance and frequency of particular words in YouTube content has the advantage of inferring public tendencies [7], especially following Zipf’s law that the top 20% of all words are used in 80% of mined text [9].

This type of analysis allows us to gain a deeper understanding into many topics, such as why nurses consider start-up businesses. According to a survey among nurses in South Korea, 85% were interested in healthcare start-ups [10]. Nurses’ motivations to launch start-ups included internal factors such as high workload, difficulties in working shifts, low empowerment, and pay [11,12], and external factors such as medical environment change due to an increase in the prevalence of chronic diseases and an aging population. These results indicate that, in the future, start-ups in the field of nursing will diversify and entrepreneurial skills will become important in the nursing field [13,14].

Entrepreneurial programs that support nurses launching start-ups are not yet active in South Korea. According to an analysis of nursing programs, only 55 out of 191 (28.8%) undergraduate nursing programs and four out of 51 (7.84%) graduate nursing programs offered business courses that would support start-ups [15]. However, there are several support initiatives available to nurses in other countries, such as the Nurses in Business Association in the United States [13,16]. These organizations could be used as examples to help build similar programs in South Korea.

Studies on nursing start-ups are limited and, to date, such studies have primarily focused on the motives or difficulties regarding nursing start-ups [15,17]. Wilson, Averis, and Walsh [18] and Johnson and Garvin [19] emphasized nursing start-ups as a field that requires experience and know-how, while Wilson, Whitaker, and Whitford [20] suggested that conducting studies which can provide practical guidance is crucial to helping nursing start-ups become successful. The field of nursing start-ups in South Korea requires extensive study and educational development programs.

Wu [21] suggested that social networks provide a variety of resources, immediate feedback, and information sharing for users to quickly acquire knowledge and obtain various educational benefits. Hence, a nursing start-up trend analysis using YouTube content will provide basic data for developing a strategy to expand nursing start-ups. It will also facilitate the development of educational content, not only in South Korea but also in various other countries. Moreover, it will be the starting point of determining various public perceptions and the current state of social trends related to nursing start-ups. The purpose of this study was to identify trends regarding nursing start-ups as reflected by YouTube content using the following methods:

1. Text mining to identify the frequency of keywords relating to nursing start-ups and their degree centrality,
2. Classifying the keywords into six categories based on Shirey’s [16] synthesis framework and a Delphi survey,
3. Comparing the results between 1) and 2) to explore trends regarding nursing start-ups.

## Materials and methods

### Study design

This study is a secondary data analysis-based survey using nursing start-up related content uploaded on YouTube.

### Theoretical framework

First, this study assumed that there is a difference between the public’s general conception of nursing start-ups and the conceptions of nurse entrepreneurs or management experts. By acknowledging this gap in perceptions, we sought to explore how the conceptions of these two parties differ and how we can utilize these differences in developing strategies that will promote nursing entrepreneurship. Therefore, in seeking a theoretical framework for this study, we examined existing gap models. We synthesized a new analysis method suitable for our study using importance-performance analysis (IPA), which involves determining priorities through a comparison of importance and performance levels, and the SERVQUAL model, which involves the establishment of marketing strategies through comparing consumer expectations and satisfaction with services. As a result, the theoretical framework of this study synthesizes a gap analysis model between a 2×2 matrix using text mining and keyword classification using a Delphi survey.

**Fig 1:**
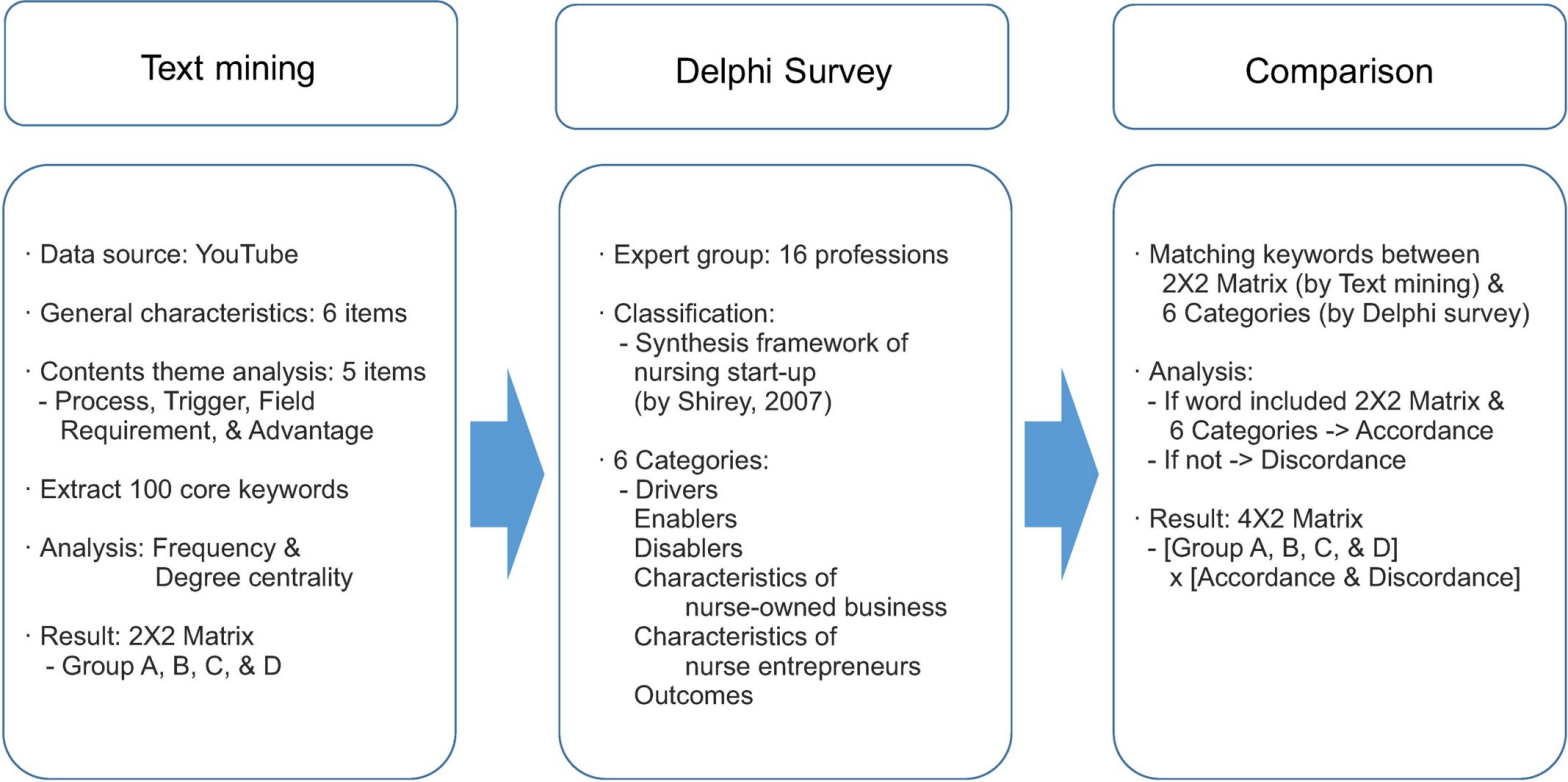
Flow of this study.

The gap analysis was conducted as follows: YouTube content related to nursing start-ups was selected. Subsequently, the 100 most frequently searched keywords were extracted for text mining. These 100 keywords were classified into four groups (A, B, C, and D) in terms of their frequency and degree centrality using a 2×2 matrix. Next, the keywords were sorted into six categories using a Delphi survey of 16 experts that followed Shirey’s [16] synthesis framework. The categorized data were subsequently divided into two groups according to their sorting consistency (50% or greater or less than 50%). Finally, to obtain information for the promotion of nursing start-ups (the goal of this study), the results of the Delphi survey were matched to the results of the 2×2 matrix from the text mining to determine which keywords showed accordance or discordance between the perspectives of the general public and experts on nursing start-ups. Keywords with high frequency and degree centrality in the text mining, which also had 50% or higher consistency in the Delphi survey, were considered core keywords of nursing start-ups according to both the general public and experts. In contrast, words of high frequency and degree centrality in the text mining analysis which had less than 50% consistency in the Delphi survey were concepts that the general public associated with nursing start-ups, but had relatively low importance according to nurse entrepreneurs. From these results, we can identify the key concepts needed for promoting nursing start-ups in the future and derive important implications needed for developing related strategies. The diagram of this research process is as follows (Fig 1).

### Study process

This study comprised three stages: text mining, a Delphi survey, and comparison between these two methods.

### Text mining

#### Subjects

The data consisted of nursing start-up related content uploaded on YouTube.

#### Measurement

The YouTube analysis tool developed by Shapiro *et al*. [22] was used for descriptive characteristics of nursing start-up content. The tool comprised six items: upload year, length, source, format, number of views, and viewer comments. The content themes were analyzed according to four items: trigger, field, requirement, and advantage. Trigger refers to factors that inspire nursing start-ups; field refers to areas where nursing start-ups are founded; requirement refers to the elements and capabilities required for nursing start-ups; and advantage refers to the benefits obtainable from nursing start-ups.

To identify keywords related to nursing start-ups from YouTube content, we measured the frequency and degree centrality of words. High frequency words are those commonly mentioned in the videos, indicating that the word is strongly related to the topic of interest. Degree centrality refers to the measurement of the number of connections (degree) that a given node (in this case, a word) has in a network [23]; it is calculated by dividing the number of adjacent nodes by the total number of nodes (not including the central, connecting node, or word). Degree centrality determines how often a word simultaneously appears with other words. The standardized degree centrality has a value ranging from 0 to 1, with a larger value indicating a more central topic of discussion (as it is connected to a large number of neighboring nodes) [24].

#### Data collection

The data consisted of content related to nursing start-ups uploaded on YouTube. To extract relevant content, we searched YouTube (http://www.youtube.com) on July 25, 2017 using the keywords “nursing entrepreneurship,” “self-employed nursing,” and “nursing start-up.” We did not require explicit permission to use any of the videos because all the videos were accessible to the public. Data collected complied with YouTube's terms of service. Using the above keywords, we extracted the titles and links of 85 videos into an Excel file. We applied the following inclusion and exclusion criteria for selecting videos. We included videos that 1) focused on nursing content, 2) covered the topic of start-ups, and 3) were presented in English. We excluded videos that 1) did not target nursing, 2) were not related to start-ups, 3) were not in English, 4) were promotional or commercial in nature, and 5) did not have an accurate data source. Two researchers independently conducted this selection process. In the event of a disagreement, they engaged a third researcher and entered into a discussion regarding the particular video until agreement was reached. The upload date, number of views, and streaming quality were not considered.

In total, 32 videos were selected for the final analysis. Python was then used to extract words in noun form from the full text of the videos. These data were collected between December 15 and 23, 2017.

#### Data analysis

In order to identify the descriptive characteristics and main theme of the content, two researchers copied the link and text of each item to an Excel file. Independent analysis was conducted according to the inclusion and exclusion criteria from September 20 to October 30, 2017. If there was a disagreement regarding the analysis, a decision was made after discussion and agreement among all three researchers involved in this study.

A network of the 100 most frequent keywords extracted from 32 YouTube clips was created using statistical computing software R. The structure and connection strength were analyzed. The relationships between the frequency and degree centrality of the keywords were classified into two groups based on the median values and analyzed using a 2x2 matrix.

### Delphi survey

#### Subjects

The expert group for the Delphi survey consisted of 16 experts, including five nurse entrepreneurs, five nurse managers, one nursing management professor, and five master’s or doctoral students majoring in business administration. The nurse entrepreneurs were all business owners who directly operated nursing homes for elderly individuals or home nursing centers, with the help of the Korea Visiting Nurse Association. The nurse managers were all currently working at hospitals but were interested in nursing start-ups. The nursing management professor had research experience in nursing management and nursing start-ups. A graduate school of management, also recruited master’s degree or PhD students majoring in business administration if they were interested in participating in the survey. The number of experts selected for the study was determined based on research by Dalkey, Brown, and Cochran [25], who reported that 10–18 experts is an appropriate number for a Delphi survey.

#### Measurement

The expert group classified the 100 mutually exclusive most frequent keywords extracted from the text mining into one of six categories relating to nursing startups, as per Shirey’s [16] synthesis framework. These categories were: drivers, enablers, disablers, characteristics of nurse-owned businesses, characteristics of nurse entrepreneurs, and outcomes. We provided participants with an explanation of the method of classifying these 100 keywords (which included how these words were extracted and an outline of Shirey’s synthesis framework), 100 cards on which these words had been written, and envelopes labeled with the six categories of Shirey’s framework to the participants of the Delphi survey. The participants were asked to choose which category each word fit best, put the corresponding word card into the appropriate envelope, and seal the envelops once the entire sorting procedure was complete. We also recorded participants’ gender, age, education level, and work experience.

#### Data collection

The Delphi survey was conducted from February 7 to March 30, 2018. A total of 16 surveys were distributed and retrieved using direct visits or e-mails.

#### Data analysis

The data were analyzed based on the classification accordance among the experts. Frequency, percentage, mean, and standard deviation were used.

### Comparison

The 2x2 matrix results from the text mining frequency and degree centrality were compared with the classification agreement for six categories from the Delphi survey. Based on the matrix, when the words matched between the text mining and the Delphi survey, they were classified as accordance. When the words did not match, they were classified as discordance.

### Ethical considerations

For the protection of the subjects’ privacy, this study was conducted upon approval of the Inha university institutional review board (Approval number: 171010-3A). The research explanation included the purpose of the study, the data collection process, and details on survey response. Furthermore, it was explained to the subjects that no particular risk was anticipated in the course of the study and that they could withdraw at any time if they so wished. In addition, it was also explained that participation was based on voluntary written consent and subjects had the right to not participate, and there was no disadvantage to not participating.

Confirmed YouTube terms and conditions is as follows. By submitting content to YouTube, you hereby grant YouTube a worldwide, non-exclusive, royalty-free, sublicenseable and transferable license to use, reproduce, distribute, prepare derivative works of, display, and perform the content in connection with the service.

## Results

### Descriptive characteristics

A total of 17 out of the 32 videos (52.5%) analyzed were uploaded between 2015 and 2017. The mean length of each video was 9.3(±11.8) minutes and the mean number of viewers was 652(±907.1). Twenty-four of the videos analyzed came from an individual, indicating that they were content which freely expressed the opinion or experience of the maker. These were the most common videos. In terms of video format, 21 were single videos, and were therefore the most common.

Table 1 shows the descriptive analysis results of nursing start-up themed YouTube content. The most common triggers for launching nursing start-ups were dissatisfaction with work environment or a lack of authority. Many start-ups were consultants who provide health education to both patients and nursing training centers. External factors such as business skills, and personal factors such as being a risk taker were identified as requirements for nursing start-ups. The advantages associated with nursing start-ups included self-employment/management, increased income, and work satisfaction.

**Table 1:** Summary of start-up content analysis of YouTube (n = 32).

Theme	Categories	N (%)	Main findings* (Number of Citation)	
**Trigger**	Yes	6(18.8)	Dissatisfaction (2), Lack of power (2), Lack of self-fulfillment (1), Helping others (1)	
	No	26(81.3)		
**Field**	Yes	14(43.7)	Training center (4), Health consulting (4), Legal consultant (1), Staffing company (1), Private nursing service (1), Medical blog in the web (1), Health application development (1), Nursing product development (1)	
	No	18(56.3)		
**Requirement**	Yes	16(50.0)	External factor (10)	Business skill (4), Education (3), Network (2), IT skill (1)
	No	16(50.0)	Personal factor (19)	Risk taker (5), Committed (3), Passion (2), Assertiveness (2), Listen to customer (2), Creativeness (2), Planner (1), Flexibility (1), Opportunity taker (1)
**Advantage**	Yes	18(56.3)	Self-directing (7), Increased income (6), Work satisfaction (6), Use of personal skill (5), Flexible working hour & freedom (4)	
	No	14(43.8)	Quality of care (2), Rewarded for personal effort (2), Expansion of world view (2), Self-fulfillment (2)	

* Multiple count results.

### Text mining

Using the text mining method, the 100 most frequently occurring words were selected. Among the identified words, “nursing” occurred 27 times (6.1%), “business” 25 times (5.6%), “entrepreneur” 16 times (3.6%), “time” 16 times (3.6%), and “information” 15 times (3.4%).

**Fig 2:**
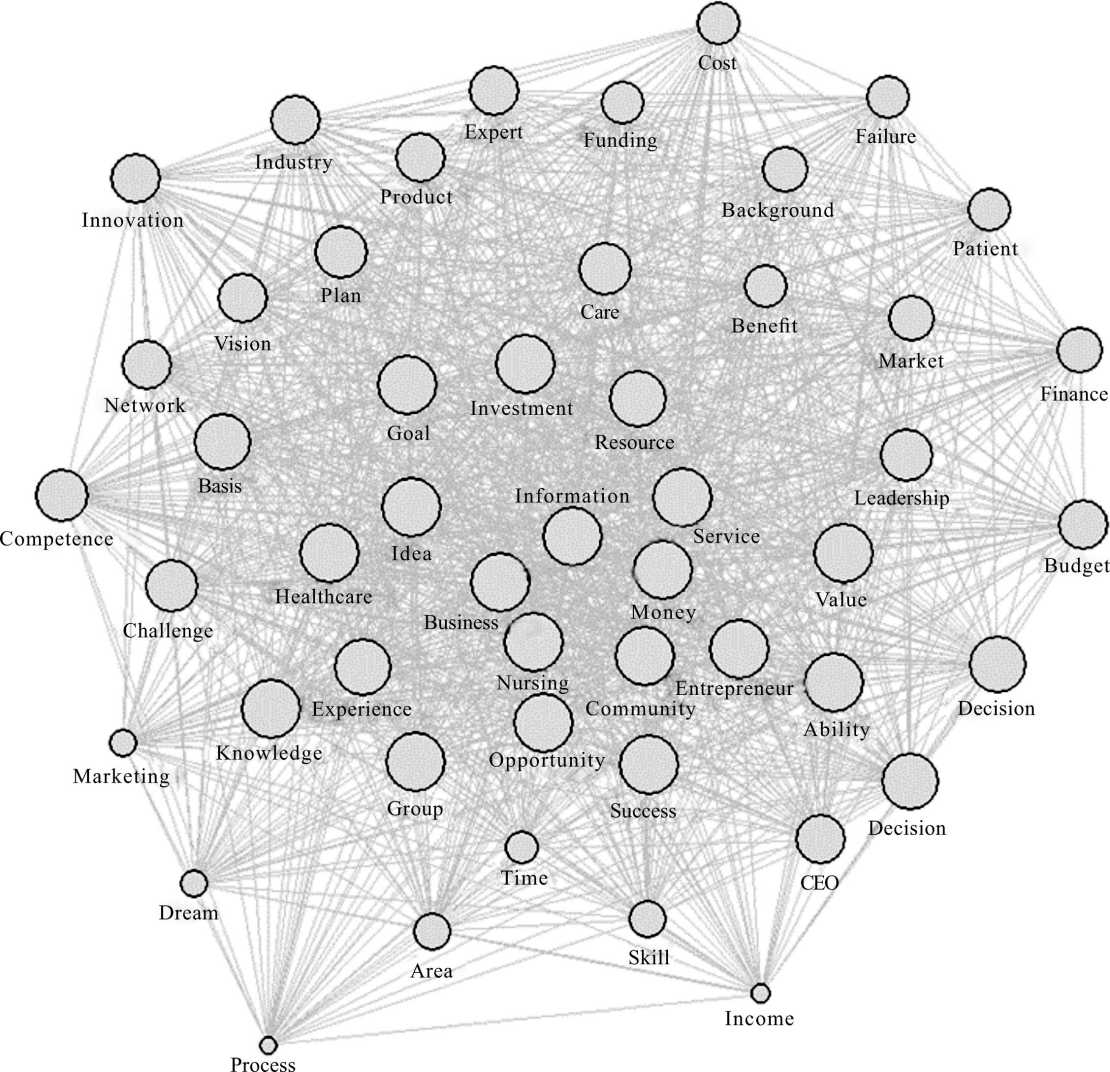
Sociogram of top 50 keywords in degree centrality.

For the visualization based on degree centrality, keywords with degree centrality over 0.5 were reclassified to create a sociogram (Fig 2). The center of the sociogram represents keywords with the highest degree centrality; “business” (0.97), “nursing” (0.96), “information” (0.89), and “money” (0.86), “service” (0.86), and “opportunity” (0.85). By contrast, the keywords located at the outer edges, representing relatively low degree centrality were “dream” (0.60), “marketing” (0.60), “income” (0.58), and “process” (0.49).

**Fig 3:**
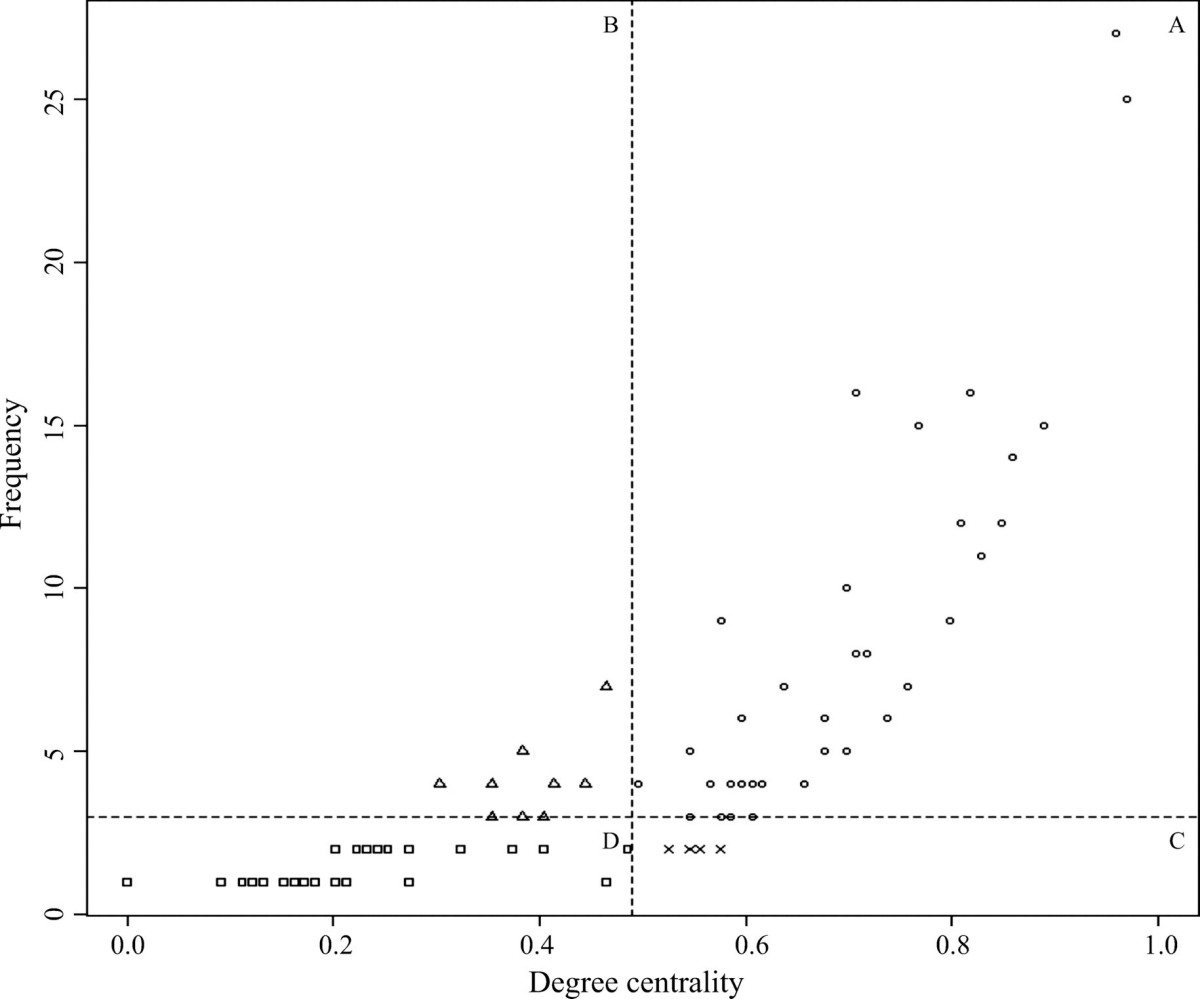
Four groups by degree centrality and frequency.

The relationship between frequency and degree centrality of the top 100 keywords was analyzed using a 2x2 matrix based on the median (Fig 3). Group A had degree centrality and frequency above the median, accounting for 42% of all words, including “area,” “business,” “competence,” “idea,” and “success.” Group B had degree centrality below the median and frequency above the median, accounting for 10% of all words, including “chance,” “consulting,” “field,” “profit,” and “training.” Group C had degree centrality above the median and frequency below the median, accounting for 8% of all words, including “budget,” “cost,” “failure,” “finance,” and “innovation.” Group D had both degree centrality and frequency below the median, accounting for 40% of all words, including “advantage,” “alternative,” “demand,” “education,” and “motivation.”

### Delphi survey

**Fig 4:**
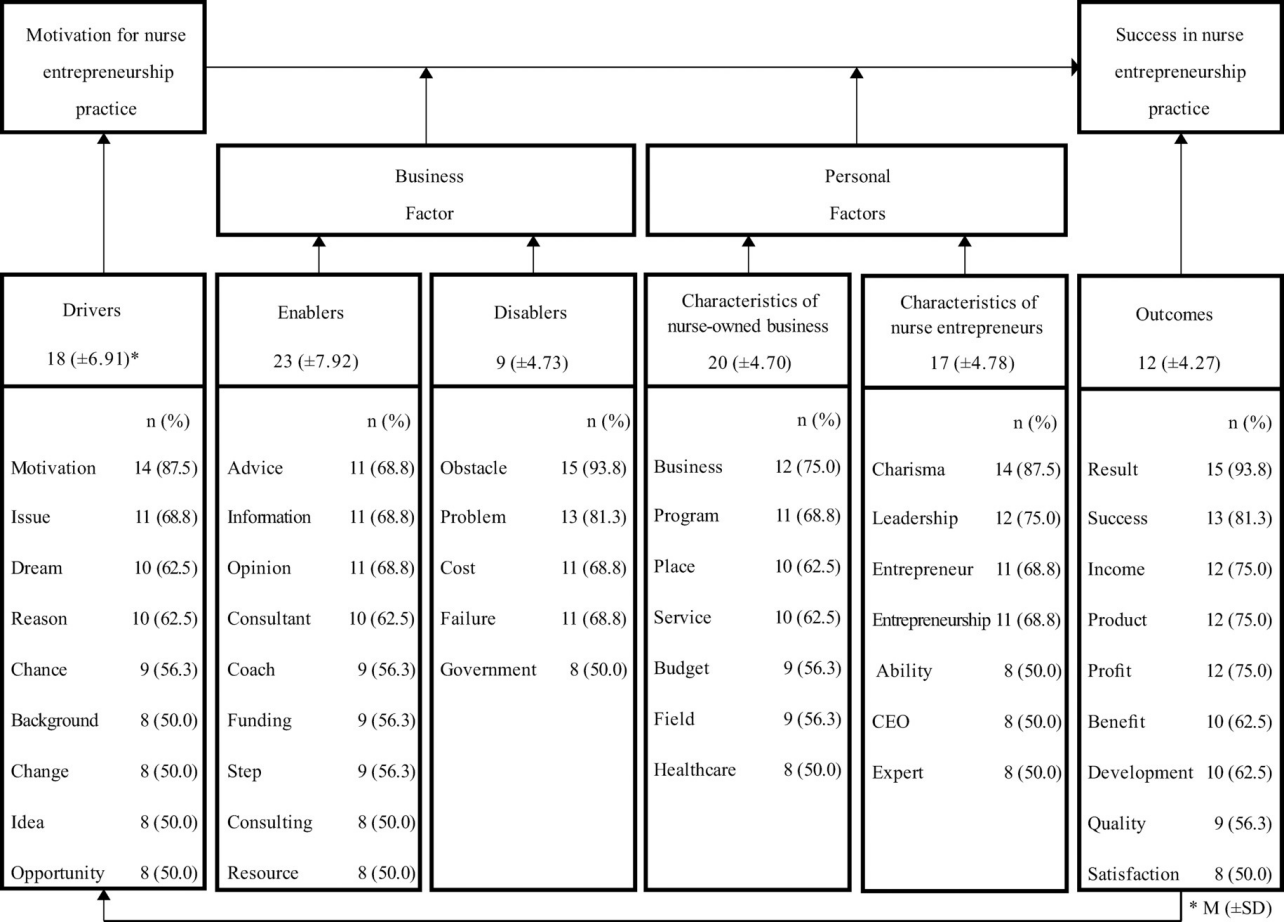
Classification of YouTube contents according to Delphi.

The following results were obtained after classifying 100 keywords related to nursing start-ups based on the six categories of Shirey’s [16] synthesis framework (Fig 4). Eighteen words (±6.91) were classified as drivers, 23 (±7.92) as enablers, 9 (±4.73) as disablers, 20 (±4.70) as characteristics of a nurse-owned business, 17 (±4.78) as characteristics of nurse entrepreneurs, and 12 (±4.27) as outcomes. The keywords that showed the highest expert group agreement in each category were “motivation” (87.5%), “advice” (68.8%), “obstacle” (93.8%), “business” (75.0%), “charisma” (87.5%), and “result” (93.8%), respectively.

### Comparison

**Fig 5:**
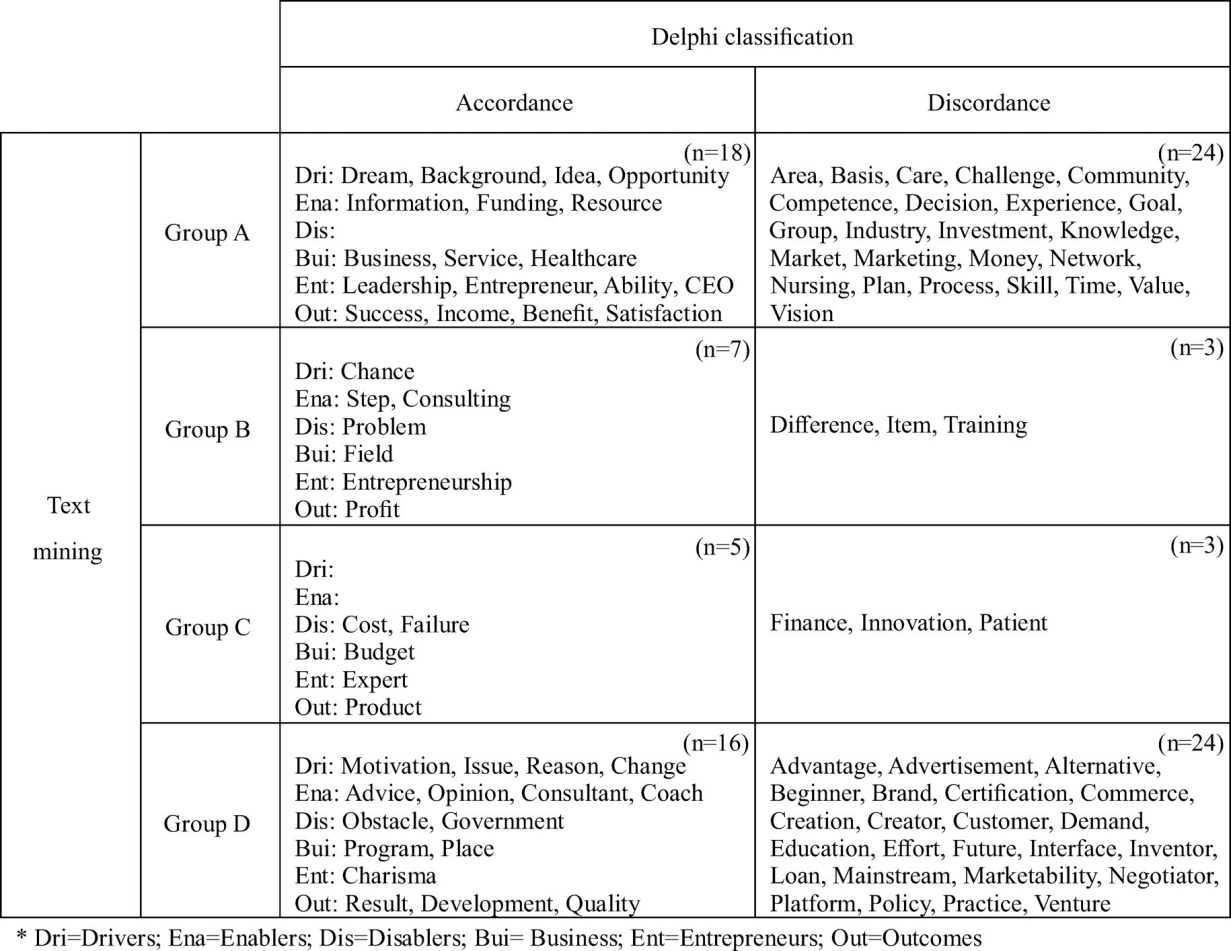
Comparison of text mining and Delphi classification.

The following results were obtained after matching the 2x2 matrix and the Delphi survey (Fig 5). For Groups A, B, C, and D, the number of words that showed accordance were 18, 7, 5, and 16, respectively, and the number that showed discordance were 24, 3, 3, and 24, respectively. Group A simultaneously showed the highest accordance as well as discordance.

## Discussion

Recently, start-ups have been emphasized as a prospective career in the field of health care [26]. In particular, a nursing start-up is a significant new outlet for women’s entrepreneurship based on professional skills [27]. In addition, startups creates economic activity for unemployed nurses and continuous career development for nurses changing jobs [28].

As a result of identifying the general characteristics of YouTube nursing start-up related content, 75% of the content identified was freely created by individuals, confirming that YouTube is a source that reflects a diverse range of opinions. Meanwhile, the quality of nursing start-up related content was not particularly high; the average length per clip was less than 10 minutes, and they were unstructured and unsystematic. These characteristics reflect how the concept of nursing start-ups is still relatively nascent.

In the content analysis, the triggers for nursing start-ups were dissatisfaction and a lack of power, which aligned with the results of previous studies showing that negative triggers act as the motivation for start-ups [11,12]. The field of nursing start-ups was mostly limited to providing education or direct nursing services for patients. Lim *et al*. [14] advocated the need for nursing service development to reflect the Fourth Industrial Revolution, such as the IoT (Internet of Things), industry convergence, and medical tourism. As a result, it has been confirmed that attempts need to be made to expand into the new field of nursing start-ups, combining the Fourth Industrial Revolution and various science and technology factors beyond the existing nursing field.

Through the Delphi survey, reclassifying keywords according to Shirey’s [16] synthesis framework led to interesting implications. Among six categories, nine keywords were classified as indicating disablers, that is, factors that make it difficult to launch a nursing start-up. However, 53 keywords were classified as indicators of drivers or enablers, which motivate nursing start-ups, and outcomes and refer to expectations from nursing start-ups. This is similar to a related literature analysis by Lim *et al*. [15]; success factors of nursing start-ups are more than just disablers. Considering that the purpose of uploading content on YouTube is to attract the attention of and provide interesting content to viewers [29], the result seems to reflect the preference for nursing start-up success cases over disturbance factors or failure cases.

Through content analysis, it was possible to infer that the government’s role in promoting nursing start-ups is not yet meeting expectations as “government” was included among the disabler keywords. Wilson *et al*. [18] reported the lack of a clear health policy, legal issues, unstructured safety nets, and lack of secure funds as disturbance factors that demonstrated the lack of government support. Major countries around the world are emphasizing the strengthening of start-ups and entrepreneurship as a survival strategy after the global financial crisis. Former President Obama of the United States introduced the Start-up America initiative as a national vision, and the European Union is pursuing programs for promoting start-ups and activating entrepreneurship [30]. Therefore, it was found that proposing policies to nurture nursing start-ups is critical in helping them succeed.

Upon matching the results of text mining and the Delphi survey, a significant concurrent trend for launching a nursing start-up was observed. First, the keywords of Group A had values higher than the median for both frequency and degree centrality, which means they are not only mentioned in a significant amount of content, but they are also core keywords agreed upon by the experts and used with other keywords. That is, the keywords in Group A reflect “main ideas” related with nursing start-ups that are widely known among interested YouTube users. The positive expectations and the public perception of nursing start-ups were identified through keywords such as “dream,” “idea,” “opportunity,” “leadership,” “success,” “benefit,” and “satisfaction” that showed accordance in Group A. Meanwhile, in terms of keywords such as “industry,” “market,” “marketing,” “money,” and “network,” which indicated discordance, we found that they should be considered as keywords that suggest the direction for growing nursing start-ups in the future because they are not yet emphasized by experts but were dealt with as important concepts in the videos. Based on this result, we expected that strengthening the content on business feasibility analysis, industrial structure, and advanced marketing strategy within existing nursing programs would lay the foundation for promoting nursing start-ups.

The keywords in Group B showed high frequency and low degree centrality, indicating they were keywords mentioned in many clips but used independently without other keywords. That is, the keywords in Group B are mentioned often and reflect “known ideas.” Words such as “entrepreneurship,” which showed accordance, were highlighted by Lim *et al*., [14] as an important concept to promote nursing start-ups.

The keywords in Group C showed low frequency and high degree centrality, which means that they were used in a few videos but with other words. That is, keywords in this group reflect “rising ideas” related to nursing start-ups that have high potential to expand in the future. “Cost,” “failure,” and “budget” showed accordance, while “finance” and “innovation” showed discordance. Through these words, it is possible to infer that it is necessary to strengthen financial and cost management in order to encourage and support nursing start-ups in the future. This finding aligned with the previous result from Cadmus *et al*., [31] which reported that strengthening financial competency through activities such as budget planning and cost accounting is the most critical issue to enhance nursing start-ups.

The keywords in Group D had values lower than the median for both frequency and degree centrality, meaning that they are not mentioned often in the content. In addition, they do not form a network with other related words. That is, the keywords in Group D are not yet clearly established in the public domain and reflect “potential ideas” that could be emphasized based on the direction that nursing start-ups take. “Motivation,” “consultant,” “charisma,” “advertisement,” “brand,” “creation,” “inventor,” “negotiator,” and “venture” suggested hidden concepts that are yet to be explored; future research will therefore be needed to develop such content aimed at encouraging future nursing start-ups.

Consequently, the trends identified in this study indicate that the concept of nursing start-ups is still in its early stage. Therefore, it is necessary to expand new nursing start-up ideas and strengthen business skills as core competencies, especially those related to finance and accounting or competitive market strategies in the Fourth Industrial Revolution. A government policy environment which supports nursing start-ups and the expansion of real nursing start-up role models can lead to the positive social perception of such start-ups. Therefore, it is necessary to discuss strategies to overcome the failures and obstacles regarding nursing start-ups through in-depth analysis. The success of a start-up depends on various factors such as government policy, science, technology, business environment, and so on. Therefore, a comprehensive nursing start-up model needs to be developed to promote nursing start-ups that include not only influential variables, but also variables that affect future changes in the start-up policy and market environment, as well as distinguish characteristics of nursing start-ups. The results of this study are expected to be utilized as a basis for the development of such a model.

## Conclusion

Content analysis of YouTube videos, categorization based on the nursing start-up model, and degree centrality using network analysis were conducted to understand the focus and importance of nursing start-ups. As a result, many keywords implying positive meanings related to drivers, enablers, and outcomes were identified while there were fewer negative keywords indicating disabling factors. Through the comparison of text mining and a Delphi survey, several key factors were found to encourage nursing startups. These include strengthening competencies in business skills such as finance and budgeting, formulating supportive government policies, and establishing new nursing start-up items appropriate to the Fourth Industrial Revolution. This study presented the characteristics and trends of nursing start-ups and will, therefore, serve as a basis for developing comprehensive nursing start-ups.

## Supporting information

S1 File List of nursing start-up related content uploaded on YouTube. (XLSX)

S2 File Questionnarie used in Delphi survey. (DOCX)
