# Supplementary material for: Identifying trends in nursing start-ups using text mining of YouTube content
Source: PLoS One. 2020 Feb 13;15(2):e0226329. doi: 10.1371/journal.pone.0226329 (PMC7018134; doi:10.1371/journal.pone.0226329)
Supplement: S2 File — (DOCX) [file pone.0226329.s002.docx]

**<A questionnaire to be used for research>**

Questionnaire on Classification of Key Languages for Nursing Startups Using Delphi Technique

| How are you?  I'm Lim Ji Young, a nursing professor at Inha University. This questionnaire is for the development of the Nurse Startup Training Program.  In order to enhance the validity and reliability of concept elicitation related to nursing start-up, we would like to hear the opinions of several teachers who are experts in nursing start-up. I would like to ask you to give me valuable feedback on the concept of a meaningful nursing start-up.  This questionnaire is intended solely for research purposes under Article 13 of the Statistics Act, and we promise not to use it for any other purpose. Also, since this questionnaire does not have a right or wrong answer, we ask for your honest and sincere answer. Participation in this study is voluntary, and you may quit even after you decide to participate, and there is no disadvantage to it. All data will be stored by researchers in a secure location with locks for three years and will be destroyed immediately thereafter. In addition, the data collected for confidentiality to individuals will be anonymized.  Thank you again for taking the time to complete the questionnaire.  October 10, 2017  Department of Nursing, Inha University  Responsible researcher: Lim Ji Young  Contact: 032-860-8210  E- mall : lim20712@inha.ac.kr  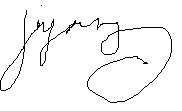 |
| --- |

※The content analysis of YouTube shows that 100 major words are among the six components of the nursing startup. A survey to categorize and assign where they belong.

● The following is a model for describing nursing start-ups presented in Shirey (2007) This nursing start-up model consists of six categories.


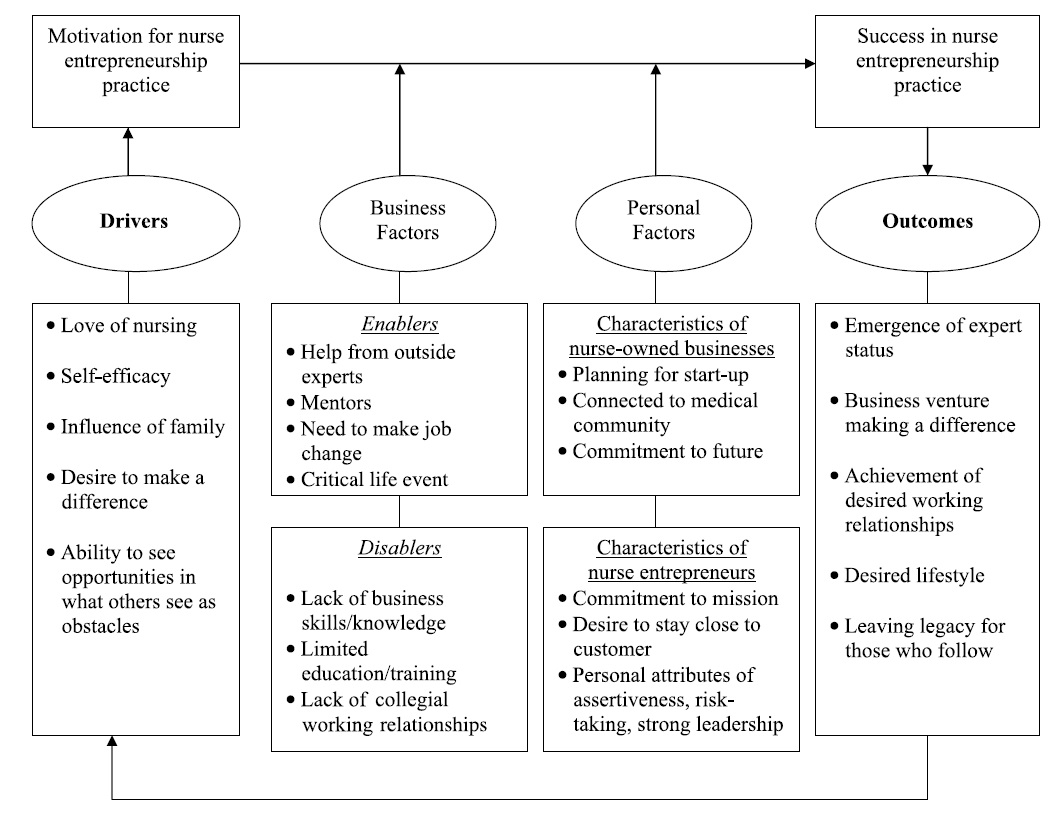


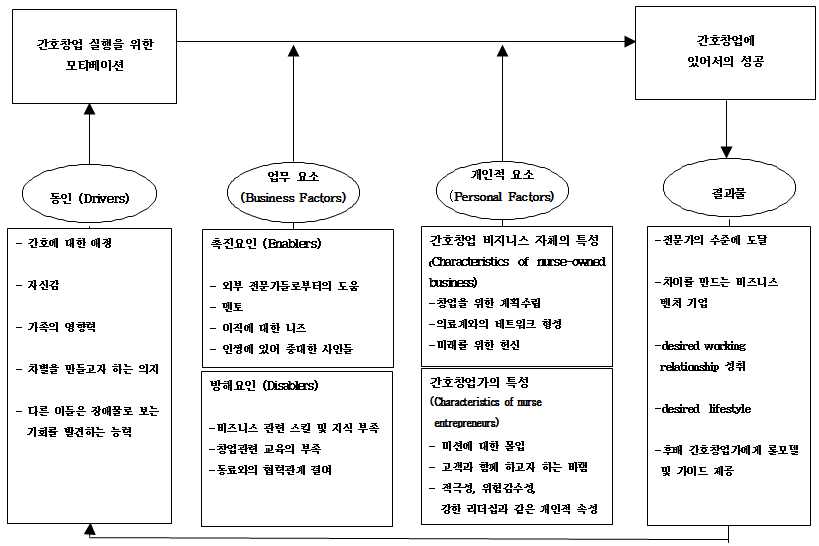


● Six key words related to nursing start-ups derived from the content analysis on YouTube Categorized and assigned as categories. The specific description of the six categories is as follows.

| **<guidebook>**  The following is a detailed description of the six sub-domain of the nursing start-up model presented by Shirey (2007). Please refer to the main language classification.  1. Drivers  Motivating nurses to pursue a spirit of start-up is concepts related to their love of nursing, their confidence as well as their family influence, their willingness to make discrimination, and their ability to see opportunities as obstacles. As a result of the research, the affection for nursing, accumulated experience and expertise enable nursing start-up work, and support of nursing entrepreneurs or families is suggested as a relevant factor.  2. Business factors-Enablers  The business component consists of two factors. Among the first facilitators is the support of mentors, help from outside experts such as lawyers, accountants and consultants. They are also important events in life, such as exhaustion of work, awareness demands such as role conflicts, childbirth or bereavement.  3. Business factors-disables  Concepts related to lack of business-related skills and knowledge required for legal, accounting or daily business operations, limited opportunities for start-up training, and lack of partnerships with colleagues.  4. Personal Factor-Characteristics of Nurse-downed business  The personal element also consists of two characteristics. The first is the notion of planning for start-up, networking with the medical community, and commitment to the future.  5. Personal Factor-Characteristics of Nurse entrepreneurs  A unique personal component of nursing entrepreneurs is the following concepts: It's the desire to be with customers, the personal nature of traditional entrepreneurs, and the concepts of activism, risk-taking, and strong leadership.  6. Outcome  The concepts related to the results of nursing start-up are as follows: Concepts such as reaching professional level, achieving good work relationships through personal and professional freedom and flexibility, providing professional role models and guides to junior nurses. |
| --- |

● The following are the top 100 keywords derived from the results of the Text mining analysis of YouTube's start-up content.

● Key words are as follows.

| **Nursing** | **Business** | **Entrepreneur** | **Success** | **Time** |
| --- | --- | --- | --- | --- |
| **Information** | **Money** | **Opportunity** | **Community** | **Service** |
| **Idea** | **Skill** | **Healthcare** | **Income** | **Group** |
| **Resource** | **Area** | **Experience** | **Training** | **Ability** |
| **Dream** | **Marketing** | **Value** | **Benefit** | **Care** |
| **Goal** | **investment** | **Knowledge** | **Step** | **Basis** |
| **CEO** | **Challenge** | **Chance** | **Consulting** | **Decision** |
| **Entrepreneurship** | **Field** | **Leadership** | **Network** | **Plan** |
| **Process** | **Vision** | **Background** | **Difference** | **Funding** |
| **Industry** | **Item** | **Market** | **Problem** | **Profit** |
| **Satisfaction** | **Brand** | **Budget** | **Competence** | **Cost** |
| **Creator** | **Customer** | **Effort** | **Expert** | **Failure** |
| **Finance** | **Innovation** | **Loan** | **Motivation** | **Obstacle** |
| **Patient** | **Product** | **Program** | **Quality** | **Venture** |
| **Advantage** | **Advertisement** | **Advice** | **Alternative** | **Beginner** |
| **Certification** | **Change** | **Charisma** | **Coach** | **Commerce** |
| **Consultant** | **Creation** | **Demand** | **Development** | **Education** |
| **Future** | **Government** | **Interface** | **Inventor** | **Issue** |
| **Mainstream** | **Marketability** | **Negotiator** | **Opinion** | **Place** |
| **Platform** | **Policy** | **Practice** | **Reason** | **Result** |

● Please classify all 100 major word cards into 6 categories presented in the model and put them in an envelope containing each component.

● Briefly describe the reason and reason for the classification by category.

● If there are six categories of words that you think are not suitable, please write the reason for the instructions by categorizing them as etc.

**※ The following are questions regarding your general characteristics: Please indicate the information.**

**(For graduate student majoring in nursing management )**

| Sex: | □ Male___ | □ Female___ |  |  |
| --- | --- | --- | --- | --- |
| Age: | ___________ |  |  |  |
| Degree course: | □ Master___ | □ Doctor___ |  |  |
| Clinical experience: | ___________years |  |  |  |

**※ The following are questions regarding your general characteristics: Please indicate the information.**

**(For nursing managers and nursing management professors)**

| Sex: | □ Male___ | □ Female___ |  |  |
| --- | --- | --- | --- | --- |
| Age: | ___________ |  |  |  |
| Affiliation: | □ University___ | □ Hospital___ |  |  |
| Clinical experience: | ___________years |  |  |  |

**※ The following are questions regarding your general characteristics: Please indicate the information.**

**(For graduate student majoring in business administration)**

| Sex: | □ Male___ | □ Female___ |  |  |
| --- | --- | --- | --- | --- |
| Age: | ___________ |  |  |  |
| Affiliation: | □ University___ | □ Hospital___ |  |  |
| Work experience: | ___________years |  |  |  |

**※ The following are questions regarding your general characteristics: Please indicate the information.**

**(For nursing entrepreneur)**

| Sex: | □ Male___ | □ Female___ |  |  |
| --- | --- | --- | --- | --- |
| Age: | ___________ |  |  |  |
| Academic background: | □ Master___ | □ Doctor___ |  |  |
| Start-up experience: | ___________years |  |  |  |
